# Vonoprazan versus proton pump inhibitors in treating post-endoscopic submucosal dissection ulcers and preventing bleeding: Protocol for meta-analysis of randomized controlled trials and observational studies

**DOI:** 10.1097/MD.0000000000014381

**Published:** 2019-02-22

**Authors:** Martin, Yi Zhou, Chun-Xu Meng, Tatsuya Takagi, Yu-Shi Tian

**Affiliations:** Graduate School of Pharmaceutical Sciences, Osaka University, 1–6 Yamadaoka, Suita City, Osaka, Japan.

**Keywords:** bleeding, meta-analysis, proton pump inhibitors, Systematic review, ulcer, vonoprazan

## Abstract

**Background:**

Vonoprazan is a potassium-competitive acid blocker (P-CAB). It is often used in Japan for *Helicobacter pylori* (*H pylori*) eradication, gastroesophageal reflux disease, and endoscopic submucosal dissection (ESD) ulcers and bleeding. This meta-analysis aims to evaluate whether vonoprazan has better therapeutic effect on ESD-induced ulcers and bleeding than proton pump inhibitors (PPIs) at different length of treatment periods (2, 4, and 8 weeks).

**Methods:**

This meta-analysis will include both randomized controlled trials (RCTs) and observational studies discussing the effectiveness of vonoprazan and PPIs on ESD-induced ulcers and bleeding. Information of studies will be collected from PubMed, Cochrane Library, ClinicalTrials.gov, and Google Scholar. Studies will be selected according to the eligibility criteria and data will be extracted by 2 people and compared with each other to keep in consistency. Cochrane risk of bias tool will be used to assess RCTs and the Newcastle-Ottawa Quality Assessment Scale will be used to assess the observational studies. Meta-analysis based on the random-effects model will be conducted to compare the differences of ulcers’ shrinkage ratios (%) and the odds ratios (OR) of scars’ stages and delayed bleeding. Publication bias will be evaluated using funnel plots and Egger's regression test. Heterogeneity will be assessed with the *I*^*2*^ statistics. Sensitivity analysis will be conducted on follow-up periods. The evidential quality of the findings will be assessed with the Grading of Recommendations Assessment Development and Evaluation (GRADE) profiler.

**Discussion:**

The findings of the present systematic review will be critical for physicians, patients, and policymakers regarding the use of vonoprazan in ESD-induced ulcers.

**Study registration:**

PROSPERO registration number: CRD42018116855.

## Abstract 2

Supplemental Digital Content is available in the text

## 1 Introduction

In Japan, the endoscopic submucosal dissection (ESD) has been considered as a preferred surgical procedure for treatment of early gastric cancer.^[1–5]^ Notably, ESD tends to induce large iatrogenic artificial ulcers and delayed bleeding complication.^[1]^ Aiming at the ESD-induced ulcers and its complication, proton pump inhibitors (PPIs) are widely prescribed after ESD.^[6]^ In December 2014, vonoprazan (TAK-438), a novel potassium-competitive acid blocker (P-CAB), was approved for clinical use in Japan.^[7]^ The P-CABs inhibit the proton pump enzyme in a reversible and potassium-competitive manner,^[8]^ resulting in a more rapid, potent, and sustained acid inhibitory effect than PPIs.^[9]^ In the preclinical trials, P-CABs were found unaffected by the time of meals and CYP2C19 polymorphism.^[10]^ All the advantages have made vonoprazan into the substitute of PPIs as the 1st-line therapy in eradicating *Helicobacter pylori* (*H pylori*).^[11–13]^

Some clinical trials and observational studies have compared the effectiveness between vonoprazan and PPIs in curing ESD-induced ulcer's scars and preventing delayed bleeding during 2 weeks,^[14,15]^ 4 weeks,^[14,16–22]^ and 8 weeks.^[14,16–18,20,23–25]^ However, the results of the effectiveness are inconsistent and controversial. One clinical trial^[20]^ reported that most ESD-induced ulcers had already healed at 4-week follow-up and implied that the evaluation of drugs at 2 weeks was more crucial. The only meta-analysis^[26]^ had selection bias in controversial studies and did not carry out an analysis of 2 weeks’ evaluation.^[26]^ Moreover, 2 clinical trials^[18,24]^ have been published after the systematic review. Thus, it still remains unclear whether vonoprazan is superior to PPIs in healing ESD-induced ulcers and preventing delayed bleeding, especially at 2-week follow-up. The present meta-analysis aims to evaluate the effectiveness of vonoprazan versus PPIs on the treatment of post-ESD ulcers and prevention of delayed bleeding complication.

## 2 Methods and analyses

This protocol is reported according to the Preferred Reporting Items for Systematic review and Meta-Analysis Protocols (PRISMA-P) 2015 checklist.^[27]^ In this protocol, the systematic review and meta-analysis was designed in accordance with the Preferred Reporting Items for Systematic Reviews and Meta-Analyses (PRISMA) guidelines.^[28]^ The protocol of this systematic review and meta-analysis has been registered in the international prospective register of systematic reviews (PROSPERO) database, with an identifier CRD42018116855.

### 2.1 Data sources and search strategies

**Figure 1:**
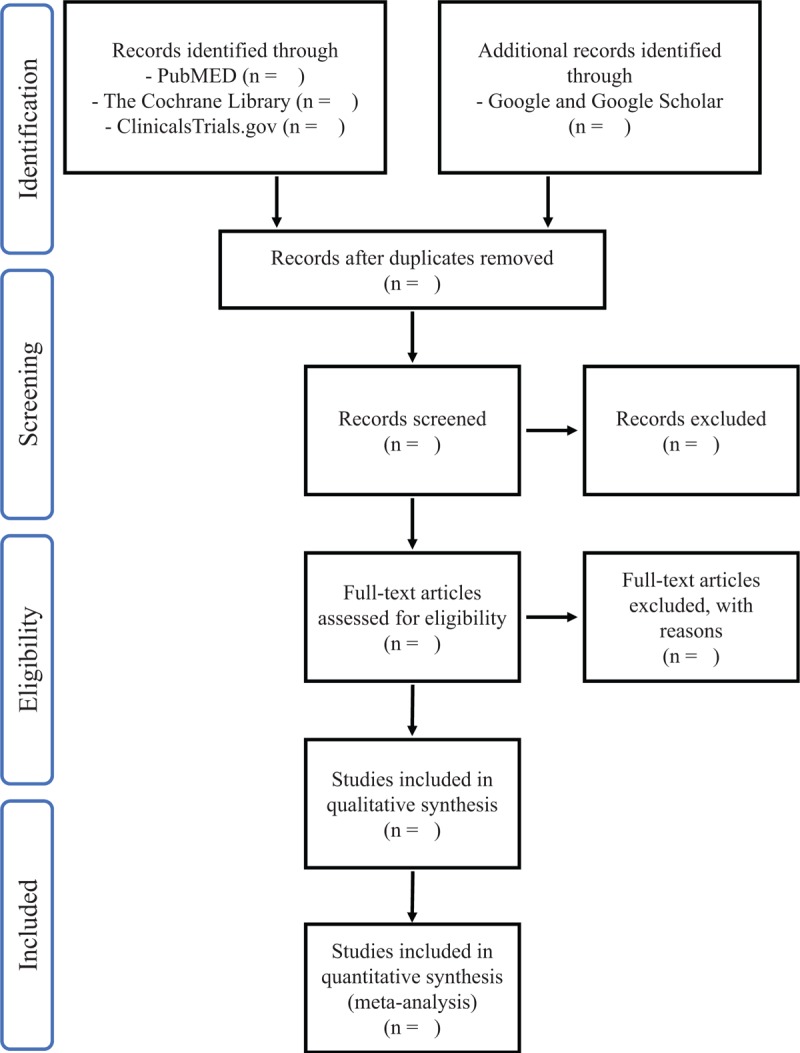
PRISMA flow diagram. PRISMA = Preferred Reporting Items for Systematic review and Meta-Analysis.

An extensive search will be conducted in PubMed, Cochrane Library, ClinicalTrials.gov, and Google Scholar. The search strategy will include keywords “Vonoprazan”, “TAK-438”, and “ESD” (Appendix 1). As for unclear reported data, further contact with the original authors will be conducted. Study selection will be conducted in a PRISMA-compliant flow chart (Fig. 1).

### 2.2 Eligibility criteria

The eligible studies will be selected in accordance with the checklist in Appendix 2 and the eligibility criteria were as follows:

#### 2.2.1 Study design

Randomized controlled trials (RCTs) and observational studies, such as cohort and case control studies, will be included. Case reports, review articles, preclinical studies, and any other non-relevant studies will be excluded.

#### 2.2.2 Follow-up periods

In order to observe changes in the ESD-induced ulcer's shrinkage rates or scar stages from the early phase, this meta-analysis will include the RCTs and observational studies with follow-up ≥ 2 weeks.

#### 2.2.3 Participants

This meta-analysis will include only the RCTs and observational studies on ESD-induced ulcer patients aged ≥ 18 years.

#### 2.2.4 Interventions

This meta-analysis will include only the RCTs and observational studies on vonoprazan monotherapy or vonoprazan combined with mucosal protective agent therapy.

#### 2.2.5 Comparators

This meta-analysis will include only the RCTs and observational studies employing PPIs monotherapy or PPIs combined with mucosal protective agent therapy as comparators.

#### 2.2.6 Outcomes

This meta-analysis will include only the RCTs and observational studies measuring shrinkage rates of ESD-induced ulcer, the number of people in ESD-induced scar stage, and the number of people with delayed bleeding. The RCTs without all these 3 outcomes will be excluded. Shrinkage rate was defined as: (1 – ulcer size at 2, 4, or 8 weeks after ESD ÷ initial ulcer size) × 100(%). Scar stage includes: Firstly, S1 and S2 stage ulcer in accordance with Sakita-Miwa classification^[29]^ or secondly, at least 90% shrinkage rate at 4-week follow-up and 100% shrinkage rate at 8-week follow-up. Delayed bleeding was defined as the decline in the hemoglobin level ≥2 g/dL.

### 2.3 Study selection

Two reviewers will use the same eligibility evaluation form to evaluate the RCTs and observational studies. Disagreement of their evaluation will be discussed with the help of a third investigator.

### 2.4 Data extraction

Data will be extracted by 1 reviewer and verified by another. In addition to the outcomes, the characteristics will be extracted as follows: First, authors and publication year. Second, interventions (dose of vonoprazan, PPIs, and mucosal protective agent). Third, baseline characteristics of participants. Fourth, follow-up periods. Fifth, study designs. Sixth, findings. The extracted data will be displayed in Appendix 3 for further analysis.

### 2.5 Quality assessment

The Cochrane risk of bias tool^[30]^ (Appendix 4) will be used to assess the design, conduction, and outcomes of the included RCTs. The Newcastle-Ottawa Quality Assessment Scale^[31]^ (Appendix 5) will be used to assess the selection, comparability, and outcome of the observational studies. The quality of each study will be assessed by 1 reviewer and verified by another. The quality of evidence will be determined with the Grading of Recommendations Assessment, Development and Evaluation (GRADE) system.^[32]^ The analysis will be conducted with GRADE profiler.

### 2.6 Data synthesis and analysis

This meta-analysis based on the random-effects model will be conducted by using RevMan version 5.3.^[33]^ For shrinkage rates, the effect will be measured as mean difference with 95% confidence intervals (CIs). Outcomes such as the number of patients in scar stage and delayed bleeding will be presented as OR with 95% CIs. Funnel plots and Egger's regression test will be implemented for publication bias evaluation. The *I*^*2*^ statistic, which describes the variations across trials rather than to sampling errors, will be calculated for heterogeneity assessment. The *I*^*2*^ values of 25, 50, and 75% indicated low, medium, and high heterogeneity. Statistical significance will be set at *P* < .05 for all analyses.

### 2.7 Sensitivity analysis

To evaluate the robustness of this meta-analysis results, sensitivity analysis will be performed by excluding the trials with mucosal protective agent combined therapy and high risk of bias (if any). We would claim this meta-analysis to be robust or reliable if the sensitivity analysis has no significant change from the results.

### 2.8 Ethical issues

This meta-analysis is a literature study, and there is no direct contact with patients. Therefore, this study does not require ethical issues approval.

## 3 Discussion

This meta-analysis will discuss the effectiveness of vonoprazan versus PPIs on curing ESD-induced ulcers and preventing delayed bleeding at different follow-up periods, especially at 2-week follow-up. The evidence would be useful for clinicians, patients, and policy-makers regarding the treatment of ESD-induced ulcers and prevention of post-ESD bleeding.

## Acknowledgments

Yi Zhou is receiving the China Government Scholarship (Award number: 201708050090) from China Scholarship Council.

## Author contributions

M and YZ conceived the study, developed the criteria and searched the literature. YZ and CXM assisted in protocol design, managed the literature, selected the studies, performed data analysis. M and YZ wrote this protocol. YST and TT advised on protocol design and revised the manuscript. All authors read and approved the final manuscript.

**Conceptualization:** Martin -, Yi Zhou.

**Data curation:** Martin -, Yi Zhou, Chun-Xu Meng.

**Methodology:** Chun-Xu Meng.

**Project administration:** Yi Zhou.

**Writing – original draft:** Martin -, Yi Zhou.

**Writing – review & editing:** Tatsuya Takagi, Yu-Shi Tian.

Tatsuya Takagi orcid: 0000-0002-0044-0722.

Yu-Shi Tian orcid: 0000-0002-8988-9453.

Yi Zhou orcid: 0000-0001-9254-3245.

## Supplementary Material

Supplemental Digital Content
