# Supplementary material for: Vonoprazan versus proton pump inhibitors in treating post-endoscopic submucosal dissection ulcers and preventing bleeding: Protocol for meta-analysis of randomized controlled trials and observational studies
Source: Medicine (Baltimore). 2019 Feb 22;98(8):e14381. doi: 10.1097/MD.0000000000014381 (PMC6408105; doi:10.1097/MD.0000000000014381)
Supplement: Supplemental Digital Content [file medi-98-e14381-s001.doc]

**Appendix 1:** Search strategies

Database: PubMed

1. Vonoprazan OR TAK-438 (all fields)

2. endoscopic submucosal dissection OR ESD (all fields)

3. #1 AND #2 (all fields)

Database: the Cochrane Library

1. Vonoprazan (Title Abstract Keyword)

2. TAK-438 (Title Abstract Keyword)

3. #1 OR #2

4. endoscopic submucosal dissection (Title Abstract Keyword)

5. ESD (Title Abstract Keyword)

6. #4 OR #5

7. #3 AND #6

Database: ClinicalTrials.gov

1. Vonoprazan (Other terms)

2. TAK-438 (Other terms)

**Appendix 2:** Eligibility criteria for screening studies

1. Is this study a randomized controlled trial or observational trial?

YES _____

NO _____

UNCLEAR _____

2. Is the follow-up period of this study at least 2 weeks?

YES _____

NO _____

UNCLEAR _____

3. Are the ages of gastric neoplasm patients in the trial at least 18 years old?

YES _____

NO _____

UNCLEAR _____

4. Is the control of this study vonoprazan monotherapy or vonoprazan combined with mucosal protective agent therapy?

YES _____

NO _____

UNCLEAR _____

5. Is the experiment of this study PPI monotherapy or PPI combined with mucosal protective agent therapy?

YES _____

NO _____

UNCLEAR _____

6. Does this study include at least one of these three outcomes: ulcer scar stage, ulcer shrinkage rate, and delayed bleeding?

YES _____

NO _____

UNCLEAR _____

7. Were the outcomes of this study reported at the same time point post-ESD?

YES _____

NO _____

UNCLEAR _____

If you answer NO to any of these questions, the study will be excluded

**Appendix 3: Basic characteristics of the included studies**

| **Study, Year** | **Study design** | **Follow-up period** | **Interventions** | | **Baseline characteristics of participants** | | | | | | | | **Findings** |
| --- | --- | --- | --- | --- | --- | --- | --- | --- | --- | --- | --- | --- | --- |
|  |  |  | **Control** | **Experiment** | **N** | **Age**  **(mean ± SD or median)** | | ***H. pylori ^a^* (+/-)** | | **Male/Female** | | **Initial ulcer size** |  |
|  |  |  |  |  |  | **VPZ ^b^** | **PPI ^c^** | **VPZ** | **PPI** | **VPZ** | **PPI** |  |  |
| Study 1 |  |  |  |  |  |  |  |  |  |  |  |  |  |
| Study 2 |  |  |  |  |  |  |  |  |  |  |  |  |  |
| Study 3 |  |  |  |  |  |  |  |  |  |  |  |  |  |
| Study 4 |  |  |  |  |  |  |  |  |  |  |  |  |  |
| …… |  |  |  |  |  |  |  |  |  |  |  |  |  |
| …... |  |  |  |  |  |  |  |  |  |  |  |  |  |

^a^ *H. pylori*, *Helicobacter pylori*; ^b^ VPZ, vonoprazan; ^c^ PPI, proton pump inhibitor

**Appendix 4:** The Cochrane Collaboration’s tool for assessing risk of bias in RCTs

| **Source of bias** | **Review authors’ judgment** | **RCT 1** | **RCT 2** | **RCT 3** | **…** |
| --- | --- | --- | --- | --- | --- |
| Random sequence generation | Selection bias due to inadequate generation of a randomized sequence | low/unclear/high | low/unclear/high | low/unclear/high |  |
| Allocation concealment | Selection bias due to inadequate concealment of allocations before assignment | low/unclear/high | low/unclear/high | low/unclear/high |  |
| Blinding of participants and personnel* | Performance bias due to knowledge of the allocated interventions by participants and personnel during the study | low/unclear/high | low/unclear/high | low/unclear/high |  |
| Blinding of outcome assessment* | Detection bias due to knowledge of the allocated interventions by outcome assessment | low/unclear/high | low/unclear/high | low/unclear/high |  |
| Incomplete outcome data* | Attrition bias due to amount, nature, or handling of incomplete outcome data | low/unclear/high | low/unclear/high | low/unclear/high |  |
| Selective reporting | Reporting bias due to selective outcome reporting | low/unclear/high | low/unclear/high | low/unclear/high |  |

**Appendix 5:** Risk of bias assessment for the cohort studies (Newcastle–Ottawa Quality Assessment Scale criteria

| **Study, year** | **Selection** | | | | **Comparability** | **Outcome** | | |
| --- | --- | --- | --- | --- | --- | --- | --- | --- |
|  | **Representativeness of the exposed cohort** | **Selection of the non-exposed cohort** | **Ascertainment of exposure** | **Demonstration that outcome of interest was not present at start of study** | **Comparability of cohorts on the basis of the design or analysis** | **Assessment of outcome** | **Was follow-up long enough for outcomes to occur** | **Adequacy of follow up of cohorts** |
|  | a) truly representative of the gastric neoplasm patients who take vonoprazan after ESD *  b) somewhat representative of the gastric neoplasm patients who take vonoprazan after ESD*  c) selected group of users e.g. nurses, volunteers  d) no description of the derivation of the cohort | a) drawn from the same community as the exposed cohort*  b) drawn from a different source  c) no description of the derivation of the non-exposed cohort | a) secure record *  b) structured interview *  c) written self-report  d) no description | a) yes *  b) no | a) study controls for age and initial ulcer size *  b) study controls for any additional factor *  c) Cohorts are not comparable on the basis of the design or analysis controlled for confounders | a) independent blind assessment *  b) record linkage *  c) self-report  d) no description | a) follow-up period of this study at least two weeks *  b) no | a) complete follow up - all subjects accounted for *  b) subjects lost to follow up unlikely to introduce bias - small number lost - > 20% *  c) follow up rate < 80% and no description of those lost  d) no statement |
| Study 1 |  |  |  |  |  |  |  |  |
| Study 2 |  |  |  |  |  |  |  |  |
| Study 3 |  |  |  |  |  |  |  |  |
| Study 4 |  |  |  |  |  |  |  |  |
| … |  |  |  |  |  |  |  |  |

Note: A study can be awarded a maximum of one * for each numbered item within the Selection and Exposure/Outcome categories. A maximum of two stars can be given for Comparability
